# SIGIRR and TNFAIP3 Are Differentially Expressed in Both PBMC and TNF-α Secreting Cells of Patients With Major Depressive Disorder

**DOI:** 10.3389/fpsyt.2021.698257

**Published:** 2021-07-28

**Authors:** Chin-Chuen Lin, Tiao-Lai Huang

**Affiliations:** ^1^Department of Psychiatry, Kaohsiung Chang Gung Memorial Hospital and Chang Gung University College of Medicine, Kaohsiung, Taiwan; ^2^Genomic and Proteomic Core Laboratory, Department of Medical Research, Kaohsiung Chang Gung Memorial Hospital, Kaohsiung, Taiwan

**Keywords:** MDD, SIGIRR, TLR, TNFAIP3, TNF-α secreting cells

## Abstract

**Background:** Major depressive disorder (MDD) is associated with the activation of the immune/inflammatory system. TNF-α is associated with MDD and poor treatment response. Toll-like receptors (TLR) are responsible in innate immune response, and is associated with MDD and antidepressant response. Some negative regulators of TLR pathway such as SOCS1, TOLLIP, SIGIRR, TNFAIP3, and MyD88s, are reported to be differentially expressed in the peripheral blood samples of patients of MDD.

**Methods:** We recruited patients with MDD and healthy controls, collect their demographic data, and measured their mRNA levels of negative TLR regulators, using peripheral blood mononuclear cells (PBMC) and isolated TNF-α secreting cells. Clinical symptoms were evaluated using Halmiton Depression Rating Scale (Ham-D). Some patients were evaluated again after 4 weeks of antidepressant treatment.

**Results:** Forty-seven patients with MDD and 52 healthy controls were recruited. Between the PBMC samples of 37 MDD patients and 42 controls, mRNA levels of SOCS1, SIGIRR, TNFAIP3, and MyD88s were significantly different. Between TNF-α secreting cells of 10 MDD patients and 10 controls, mRNA levels of SIGIRR and TNFAIP3 were significantly different. Change of Ham-D score only correlated significantly with TOLLIP mRNA level after treatment.

**Conclusion:** SIGIRR and TNFAIP3, two negative regulators of TLR immune response pathways, were differentially expressed in both PBMC and TNF-α secreting cells of patients with MDD as compared to healthy controls. The negative regulations of innate immune response could contribute to the underlying mechanism of MDD.

## Introduction

Major depressive disorder (MDD) has been associated with the activation of the immune/inflammatory system, including changes in serum acute phase protein (1, 2) and cytokine levels (3–5). Antidepressant treatment has also been shown to normalize the inflammatory state, by decreasing serum levels of proinflammatory cytokines such as IL-12 and increasing serum levels of anti-inflammatory cytokines such as IL-4 and TGF-β1 (6). Increased plasma levels of IL-6 and TNF-α before treatment predicted poor antidepressant response (7, 8). A meta-analysis has shown that increased serum levels of TNF-α and IL-6 are the most replicated findings in MDD (9).

Toll-like receptors (TLRs) are the pattern recognition receptors that recognize pathogenic exogenous and endogenous molecular patterns and play an important role in the innate immune system. In humans, 10 TLRs (TLR-1 to 10) were characterized. TLR-1, 2, 4, 5, and 6 are at the cell membrane, detecting bacteria. For example, TLR-4 binds lipopolysaccharide in gram-negative bacteria. TLR-3, 7, 8, and 9 are located on intracellular endosomes, and detect nucleic acids from bacteria and viruses that have penetrated the cell. For example, TLR-7 binds single-stranded RNA from viruses as well as some endogenous proteins. After receptor binding, a cascade is initiated, leading to transcriptions of inflammatory cytokines (10). Prior studies have shown that TLR expressions were associated with MDD diagnosis (11) and depressive symptoms (12). Antidepressant treatment could normalize elevated TLR expressions prior to medications (13). In the recent years, attention has turned to negative regulators of TLR pathway, suggesting that dysfunction in the negative feedback loop could also contribute to the psychopathology of MDD (13, 14). Some of the more frequent investigated negative regulators include suppressor of cytokine signaling 1 (SOCS1), Toll-interacting protein (TOLLIP), single immunoglobulin interleukin-1-related receptor (SIGIRR), TNF-α-induced protein 3 (TNFAIP3), and the short form of MyD88 (MyD88s) (15).

Earlier studies regarding TLR expressions and their negative regulators used peripheral blood mononuclear cells (PBMC) as the analyzed sample (14), which contain a variety of cells. Recent advances in technology allowed isolation of specific types of cells, such as monocytes (16) and TNF-α secreting cells (17). As mentioned earlier, TNF-α is an important cytokine in MDD (9). Therefore, in this study, we intended to investigate the mRNA levels of five negative TLR regulators (SOCS1, TOLLIP, SIGIRR, TNFAIP3, and MyD88s) in PBMC and TNF-α secreting cells from patients of MDD, compared to the healthy controls and after antidepressant treatment.

## Materials and Methods

### Study Samples

From September 2017 to July 2018, hospitalized patients diagnosed with MDD were recruited at the Chang Gung Memorial Hospital. MDD was diagnosed by a psychiatrist according to the criteria of the *Diagnostic and Statistical Manual of Mental Disorders, Fifth Edition* (DSM-5). Only patients aged between 20 and 65 years old were included. Patients with systemic diseases, such as cardiovascular diseases, liver diseases and thyroid diseases, smokers, or patients with alcohol dependence were excluded. The severity of depression was assessed by the 17-item Halmiton Depression Rating Scale (Ham-D) (18). The choice of antidepressants depended on what the clinicians considered best for the patients. Healthy controls were recruited and assessed by semi-structured interviews to rule out psychiatric disorder according to DSM-5 criteria. Written informed consent was provided by all participants after the content and context of the study was fully explained. The institutional review board (IRB) of Chang Gung Memorial Hospital approved the study design (IRB 201602052B0C501).

### Laboratory Data

Venous blood of 15 ml was drawn from each participant in the morning following a 6-h fast. Peripheral blood mononuclear cells (PBMC) were isolated using Ficoll-Paque medium. TNF-α secreting cells were further isolated from PBMC using TNF-α Secretion Assay-Cell Enrichment and Detection Kit (Miltenyi Biotec, #130-091-269). Isolated cells were stored at −80°C until assay.

### Quantitative Reverse Transcription-Polymerase Chain Reaction Analysis

Quantitative reverse transcription-polymerase chain reaction (qRT-PCR) was performed using the following sets of primers: SOCS1 5′-GACCCCTTCTCA CCTCTTGA-3′ (sense) and 5′-GTAGGAGGTGCGAGTTCAGG-3′ (antisense); TOLLIP, 5′-GACAACTGTCTCCGTCGCA-3′ (sense) and 5′-CGGGAGCTCACCGATGTA-3′ (antisense); SIGIRR, 5′-CCCAGCTCTTGGATCAGTCT-3′ (sense) and 5′-AGTCAGGGGCCCTATCACAG-3′ (antisense); TNFAIP3, 5′-GGACTT TGCGAAAGGATC G-3′ (sense) and 5′-TCACAGCTTTCCGCATATTG-3′ (antisense); MyD88s, 5′-TCATCGAAAAGAGGTTGGCT-3′(sense) and 5′-GATGGG GATCAGTCGCTTCT-3′ (antisense); glyceraldehyde-3-phosphate dehydrogenase (GAPDH), 5′-TGCACCACC AACTGCTTAGC-3′ (sense) and 5′-GGCATGGACTGTGGTCATGAG-3′ (antisense).

The relative abundance of mRNAs was calculated with the comparative Ct method using GADPH as the housekeeping gene, represented by -ΔCt, to make comparisons with earlier studies possible. Fold changes were calculated with 2^∧^ − ΔΔCt.

### Statistical Analysis

All results are represented as mean ± standard deviation. Comparisons of study groups were calculated using independent *t*-test, Wilcoxan sign rank test, or Mann-Whitney *U*-Test. Pearson correlation was used to assess the relationship with the associated parameters. Data analysis was performed using SPSS 19 (Chicago, IL, U.S.A.). *p*-values of <0.05 were considered statistically significant.

## Results

Forty-seven patients with MDD and 52 healthy controls were recruited. Samples of 37 patients and 42 controls were analyzed for PBMC data. Thirteen patients were treated with antidepressants for 4 weeks, and their PBMC data were analyzed both at baseline and after treatment. Samples of 10 patients and 10 controls were analyzed for TNF-α secreting cells. Medications of the 37 MDD patients were summarized in Supplementary Table 1.

Between the PBMC samples of 37 MDD patients (12 males and 25 females) and 42 controls (18 males and 24 females), mRNA levels of SOCS1, SIGIRR, TNFAIP3, and MyD88s were significantly different, using independent *t*-test (*p* = 1.1 × 10^−5^, 2.0 × 10^−8^, 6.0 × 10^−14^, and 7.2 × 10^−12^, respectively). Their demographic data and mRNA levels were summarized in Table 1. Fold changes of MDD group were relative to the controls. No significant difference in GPDPH expression was found between MDD patients and controls. SOCS1 level correlated significantly with levels of TNFAIP3 and MyD88s (*p* = 0.001 and 0.014, respectively). SIGIRR level correlated significantly with BMI, Ham-D score, TNFAIP3 level, and MyD88s level (*p* = 0.026, 0.000, 0.000, and 0.000, respectively). TNFAIP3 level correlated significantly with BMI, SOCS level, SIGIRR level, and MyD88s level (*p* = 0.011, 0.001, 0.000, and 0.000, respectively). MyD88s level correlated significantly with BMI, Ham-D score, SOCS level, SIGIRR level, and TNFAIP3 level (*p* = 0.010, 0.000, 0.014, 0.000, and 0.000, respectively).

**Table 1:** Demographic data and mRNA levels of PBMC.

	**MDD (*n* = 37)**	**Controls (*n* = 42)**	**Fold change**	***p***
Age (years)	49.6 ± 13.0	42.6 ± 7.8		0.006^*^
Onset (years)	41.6 ± 14.4			
Duration (years)	8.0 ± 8.3			
BMI (kg/m^2^)	25.3 ± 4.9	23.8 ± 3.6		0.141
Ham-D score	16.4 ± 20.0			
SOCS1 (-ΔCt)	−5.9 ± 1.5	−7.2 ± 0.9	3.8 ± 3.1	1.1 × 10^−5^^*^
TOLLIP (-ΔCt)	−10.6 ± 0.9	−10.5 ± 0.5	1.2 ± 0.7	0.823
SIGIRR (-ΔCt)	−7.2 ± 1.6	−5.3 ± 1.1	0.4 ± 0.4	2.0 × 10^−8^^*^
TNFAIP3 (-ΔCt)	−7.0 ± 1.7	−4.3 ± 0.9	0.3 ± 0.30	6.0 × 10^−14^^*^
MyD88s (-ΔCt)	−9.5 ± 2.0	−6.8 ± 0.9	20.5 ± 1.8	7.2 × 10^−12^^*^

* *p < 0.05.BMI, body mass index; Ham-D, 17-item Halmiton Depression Rating Scale; MDD, major depressive disorder; MyD88s, the short form of MyD88; PBMC, peripheral blood mononuclear cells; SIGIRR, single immunoglobulin interleukin-1-related receptor; SOCS1, suppressor of cytokine signaling 1; TLR, Toll-like receptors; TNFAIP3, TNF-α-induced protein 3; TOLLIP, Toll-interacting protein*.

In the 13 treatment completers (1 male and 12 females), mRNA levels of PBMC did not differ significantly before and after treatment using Wilcoxan sign rank test, despite the significant decrease of Ham-D score. Their demographic data and mRNA levels were summarized in Table 2. Fold changes of post-treatment group were relative to the baseline. No significant difference in GPDPH expression was found before and after treatment. The demographic data of the treatment completers do not differ significantly from the 37-patient group, though the treatment completers had more severe depression at baseline. Change of Ham-D score only correlated significantly with TOLLIP mRNA level after treatment (*p* = 0.021).

**Table 2:** Demographic data and mRNA levels of PBMC before and after antidepressant treatment.

	**Baseline (*n* = 13)**	**After treatment (*n* = 13)**	**Fold change**	***p***
Age (years)	45.6 ± 15.3			
BMI (kg/m^2^)	24.1 ± 6.2	24.3 ± 6.0		0.301
Ham-D score	36.2 ± 20.7	16.2 ± 10.3		0.002^*^
SOCS1 (-ΔCt)	6.1 ± 1.2	6.4 ± 1.5	1.4 ± 1.6	0.480
TOLLIP (-ΔCt)	11.2 ± 0.7	11.2 ± 0.7	1.1 ± 0.5	0.638
SIGIRR (-ΔCt)	5.7 ± 0.8	5.8 ± 0.8	1.1 ± 0.7	0.754
TNFAIP3 (-ΔCt)	6.3 ± 1.6	6.6 ± 1.4	1.2 ± 1.0	0.239
MyD88s (-ΔCt)	7.8 ± 0.9	7.8 ± 0.9	1.2 ± 0.9	0.937

* *p < 0.05.BMI, body mass index; Ham-D, 17-item Halmiton Depression Rating Scale; MDD, major depressive disorder; MyD88s, the short form of MyD88; PBMC, peripheral blood mononuclear cells; SIGIRR, single immunoglobulin interleukin-1-related receptor; SOCS1, suppressor of cytokine signaling 1; TLR, Toll-like receptors; TNFAIP3, TNF-α-induced protein 3; TOLLIP, Toll-interacting protein*.

Between TNF-α secreting cells of 10 MDD patients (1 male and 9 females) and 10 controls (2 males and 8 females), mRNA levels of SIGIRR and TNFAIP3 were significantly different, using Mann-Whitney U Test (*p* = 0.023 and 3.2 × 10^−4^, respectively). Their demographic data and mRNA levels were summarized in Table 3. Fold changes of MDD group were relative to the controls. No significant difference in GPDPH expression was found between MDD patients and controls. SIGIRR level correlated significantly with SOCS1 level and TNFAIP3 level (*p* = 0.000 and 0.001, respectively). TNFAIP3 level correlated significantly with SOCS1 level, SIGIRR level, and MyD88s level (*p* = 0.042, 0.001, and 0.004, respectively).

**Table 3:** Demographic data and mRNA levels from TNF-α secreting cells.

	**MDD (*n* = 10)**	**Controls (*n* = 10)**	**Fold change**	***p***
Age (years)	44.5 ± 10.8	42.3 ± 9.4		0.634
Onset (years)	37.2 ± 13.0			
Duration (years)	7.4 ± 8.2			
BMI (kg/m^2^)	23.6 ± 2.9	24.2 ± 3.6		0.678
Ham-D score	14.2 ± 14.9			
SOCS1 (-ΔCt)	6.9 ± 0.6	6.0 ± 1.0	0.6 ± 0.2	0.063
TOLLIP (-ΔCt)	10.9 ± 0.7	11.4 ± 0.5	1.5 ± 0.6	0.089
SIGIRR (-ΔCt)	7.6 ± 0.3	5.6 ± 2.1	0.2 ± 0.5	0.023^*^
TNFAIP3 (-ΔCt)	0.50 ± 0.4	0.7 ± 0.7	2.4 ± 0.7	3.2 × 10^−4^^*^
MyD88s (-ΔCt)	6.7 ± 0.7	7.0 ± 0.7	21.8 ± 1.5	0.631

* *p < 0.05*.

*BMI, body mass index; Ham-D, 17-item Halmiton Depression Rating Scale; MDD, major depressive disorder; MyD88s, the short form of MyD88; SIGIRR, single immunoglobulin interleukin-1-related receptor; SOCS1, suppressor of cytokine signaling 1; TLR, Toll-like receptors; TNFAIP3, TNF-α-induced protein 3; TOLLIP, Toll-interacting protein*.

## Discussion

The most important finding of this study is that SIGIRR and TNFAIP3, two negative regulators of TLR immune response pathways, were differentially expressed in both PBMC and TNF-α secreting cells of patients with MDD as compared to healthy controls. While both SIGIRR and TNFAIP3 mRNA levels had been investigated in the PBMC of patients with MDD in the past (14), only TNFAIP3 mRNA level was reported in TNF-α secreting cells of patients with MDD before (17). To our knowledge, this is the first study to report the differential expression of SIGIRR in TNF-α secreting cells of patients with MDD as compared to healthy controls.

SIGIRR is a transmembrane TLR regulator, which binds to TLR4 and interleukin-1 receptor associated kinase (IRAK) to inhibit the downstream TLR signal pathways (15). In this study, SIGIRR were significantly lower in patients in MDD whether the samples were PBMC or TNF-α secreting cells. Previously, lower SIGIRR was found in PBMC in patients with MDD, but not statistically significant (14). In another study analyzing SIGIRR in monocyte sample of patients of MDD, significantly higher level of SIGIRR was found (16). We speculate that the lower SIGIRR in PBMC and TNF-α secreting cells indicate a deficiency to prevent TLR inflammation. Given most of the studies, including our own, had limited sample size, further investigations would be needed to confirm the significance of SIGIRR in TLR regulation in MDD.

TNFAIP3, also known as A20, is an intracellular TLR regulator, which deubiquitylates tumor-necrosis factor-receptor-associated factor 6 (TRAF6), thus inhibiting the downstream activation of NF-κB inflammatory pathway (15). TNFAIP3 is a potent regulator of dendritic spine remodeling and synapse efficacy in neurons (19). TNFAIP3 had been investigated in various samples from patients of MDD in the past, including PBMC, monocytes, and TNF-α secreting cells (14, 16, 17). In this finding, we found significantly lower TNFAIP3 mRNA level in patients with MDD, which was in line with earlier studies on PBMC (14) and monocytes (16). However, in the TNF-α secreting cells, we found significantly higher mRNA levels of TNFAIP3 in patients of MDD, while an earlier study also found higher TNFAIP levels, no statistical significance was found (17). While lower TNFAIP3 levels in PBMC and monocytes in patients of MDD could indicate a failed defense against TLR inflammation, the higher level found in TNF-α secreting cells in this study might also be an exaggerated response from the overall inflammatory state in patients of MDD. There had also been reports of abnormalities of TNFAIP3 in other psychiatric disorders. In the PBMC of adolescents diagnosed bipolar I disorder, TNFAIP3 mRNA level correlated with pediatric inpatient aggression prediction score, as well as functional brain activations of right anterior part of anterior cingulate gyrus, a part of aggression pathway (20). Significantly higher levels TNFAIP3 mRNA levels were found in the monocytes of patients of bipolar disorder (21). The abnormalities of TLR pathway negative regulators could also be found in psychiatric disorders other than MDD.

We also found SOCS1 to be statistically higher and MyD88s to be statistically lower in PBMC of patients of MDD, though no statistical significance was found in TNF-α secreting cells. SOCS1 suppresses IRAK to prevent inflammatory response initiated by TLR 4 and 9 (15, 22, 23). In our study, significantly higher SOCS1 level was found in PBMC of patients with MDD. Previously, significantly higher SOCS1 was also found in the monocytes of patients of MDD (16), but not in PBMC (14). SOCS1 had also been investigated in other mood disorder, namely bipolar disorder. SOCS1 mRNA levels were also significantly higher in patients with bipolar disorder, but this finding remained positive in male patients only if different genders were analyzed separately (24). SOCS1 could also contribute to the innate immune responses associated with MDD.

MyD88s is the short form of MyD88, which is the most crucial adaptor in TLR signaling (15). MyD88s antagonizes MyD88 functions, preventing IRAK4 to phosphorylate IRAK1, thus halting the inflammatory pathway. In our study, we found significantly lower MyD88s mRNA levels in the PBMC in patients with MDD, similar to past findings in PBMC (14) and monocytes (16). The negative regulators of TLR signaling could form a complex web, and further investigations are warranted. The recent findings of negative TLR regulators of MDD are summarized in Table 4.

**Table 4:** Summary of recent findings of negative regulators of TLR pathway (-ΔCt).

**Publication**	**Sample cells**	**MDD vs. controls**	**SOCS1**	**TOLLIP**	**SIGIRR**	**TNFAIP3**	**MyD88s**
Hung 2017	PBMC	100 vs. 53	↓	↑	↓	↓^*^	↓^*^
Hung 2018	monocytes	34 vs. 33	↑^*^	↓^*^	↑^*^	↓^*^	↓^*^
Huang 2019	TNF-α secreting cells	30 vs. 30				↑	
Present study	PBMC	37 vs. 42	↑^*^	↓	↓^*^	↓^*^	↓^*^
	TNF-α secreting cells	10 vs. 10	↓	↑	↓^*^	↑^*^	↑

* *p < 0.05*.

*MDD, major depressive disorder; MyD88s, the short form of MyD88; SIGIRR, single immunoglobulin interleukin-1-related receptor; SOCS1, suppressor of cytokine signaling 1; TLR, Toll-like receptors; TNFAIP3, TNF-α-induced protein 3; TOLLIP, Toll-interacting protein. Up arrow refers to the values of MDD are greater than those of controls, and vice versa*.

In the patients treated with antidepressants for 4 weeks, we did not find significant changes in the levels of those negative regulator mRNA. Earlier studies could not find significant changes in levels of SOCS1, TOLLIP, SIGIRR, or MyD88s in PBMC or monocytes of patients with MDD, but TNFAIP3 levels showed significant increase after treatment (14, 16). TNFAIP3 level is also associated with psychological anxiety in MDD (25), baseline Ham-D score (14), and decreases in Ham-D score (14). While in another study, SOCS1 level correlated with changes in Ham-D score (16). In our study, only TOLLIP mRNA levels after treatment correlated significantly with changes in Ham-D score. The exact mechanism of how those regulators contribute to clinical symptoms remained elusive.

There are several limitations in this study. The sample sizes of both PBMC and TNF-α secreting cells investigations were small. The antidepressants were not controlled. Different classes of antidepressants could interact with the targeted outcomes in various ways. The treatment duration was merely 4 weeks, which is relatively short compared to other studies involving antidepressant treatment, which usually lasted 8–12 weeks. Due to limited fund, we were unable to analyze negative TLR regulators of TNF-α secreting cells after antidepressant treatment. Lastly, TNF-α-secreting cells include several cell types, such as monocytes, macrophages, and T cells, which could confound the findings. The readers are warned against over-interpret our study results because of those limitations, and a larger sample size with more controlled variables will be needed before a firm conclusion could be made.

SIGIRR and TNFAIP3, two negative regulators of TLR immune response pathways, were differentially expressed in both PBMC and TNF-α secreting cells of patients with MDD as compared to healthy controls. The negative regulations of innate immune response could contribute to the underlying mechanism of MDD.

## Data Availability Statement

The raw data supporting the conclusions of this article will be made available by the authors, without undue reservation.

## Ethics Statement

The studies involving human participants were reviewed and approved by Institutional Review Board of Chang Gung Memorial Hospital. The patients/participants provided their written informed consent to participate in this study.

## Author Contributions

T-LH contributed substantially to conception, design, and approved the final draft. C-CL and T-LH contributed to acqusition of data, helped with analysis, and interpretation of data. C-CL drafted the article and revised it critically for important intellectual content. All authors contributed to the article and approved the submitted version.

## Conflict of Interest

The authors declare that the research was conducted in the absence of any commercial or financial relationships that could be construed as a potential conflict of interest.

## Publisher's Note

All claims expressed in this article are solely those of the authors and do not necessarily represent those of their affiliated organizations, or those of the publisher, the editors and the reviewers. Any product that may be evaluated in this article, or claim that may be made by its manufacturer, is not guaranteed or endorsed by the publisher.
