# Supplementary material for: SIGIRR and TNFAIP3 Are Differentially Expressed in Both PBMC and TNF-α Secreting Cells of Patients With Major Depressive Disorder
Source: Front Psychiatry. 2021 Jul 28;12:698257. doi: 10.3389/fpsyt.2021.698257 (PMC8356912; doi:10.3389/fpsyt.2021.698257)
Supplement: Supplementary file 1 [file Table_1.pdf]

Supplementary Table 1. Medications used by patients with MDD

Category	Name	Number (%)	Categorical total
SSRI	Paroxetine	11 (29.7%)	21 (56.8%)
	Fluoxetine	6 (16.2%)	
	Escitalopram	4 (10.8%)	
SNRI	Duloxetine	6 (16.2%)	9 (24.3%)
	Venlafaxine	3 (8.1%)	
Misc.	Mirtazapine	2 (5.4%)	7 (18.9%)
	Lithium	2 (5.4%)	
	Trazodone	1 (2.7%)	
	Agomelatine	1 (2.7%)	
	Valproate	1 (2.7%)	

MDD: major depressive disorder; SNRI: serotonin–norepinephrine reuptake inhibitor; SSRI: selective serotonin reuptake inhibitor
